# Frequent associations between CTL and T-Helper epitopes in HIV-1 genomes and implications for multi-epitope vaccine designs

**DOI:** 10.1186/1471-2180-10-212

**Published:** 2010-08-09

**Authors:** Sinu Paul, Helen Piontkivska

**Affiliations:** 1 Department of Biological Sciences, Kent State University, Kent, Ohio, 44242, USA

## Abstract

**Background:**

Epitope vaccines have been suggested as a strategy to counteract viral escape and development of drug resistance. Multiple studies have shown that Cytotoxic T-Lymphocyte (CTL) and T-Helper (Th) epitopes can generate strong immune responses in Human Immunodeficiency Virus (HIV-1). However, not much is known about the relationship among different types of HIV epitopes, particularly those epitopes that can be considered potential candidates for inclusion in the multi-epitope vaccines.

**Results:**

In this study we used association rule mining to examine relationship between different types of epitopes (CTL, Th and antibody epitopes) from nine protein-coding HIV-1 genes to identify strong associations as potent multi-epitope vaccine candidates. Our results revealed 137 association rules that were consistently present in the majority of reference and non-reference HIV-1 genomes and included epitopes of two different types (CTL and Th) from three different genes (*Gag, Pol *and *Nef*). These rules involved 14 non-overlapping epitope regions that frequently co-occurred despite high mutation and recombination rates, including in genomes of circulating recombinant forms. These epitope regions were also highly conserved at both the amino acid and nucleotide levels indicating strong purifying selection driven by functional and/or structural constraints and hence, the diminished likelihood of successful escape mutations.

**Conclusions:**

Our results provide a comprehensive systematic survey of CTL, Th and Ab epitopes that are both highly conserved and co-occur together among all subtypes of HIV-1, including circulating recombinant forms. Several co-occurring epitope combinations were identified as potent candidates for inclusion in multi-epitope vaccines, including epitopes that are immuno-responsive to different arms of the host immune machinery and can enable stronger and more efficient immune responses, similar to responses achieved with adjuvant therapies. Signature of strong purifying selection acting at the nucleotide level of the associated epitopes indicates that these regions are functionally critical, although the exact reasons behind such sequence conservation remain to be elucidated.

## Background

Human Immunodeficiency Virus (HIV), the virus responsible for Acquired Immunodeficiency Syndrome (AIDS), is one of the major causes of death around the world today. There were 2.1 million AIDS related deaths and 2.5 million new infections in 2007 alone with over 33.2 million people living with HIV-1 infection (AIDS epidemic update 2007, UNAIDS). Although the use of the Highly Active Anti-Retroviral Therapy (HAART) has significantly reduced the mortality and morbidity of HIV patients by chronically suppressing HIV-1 replication, we are far from finding a cure [1,2]. Moreover, drug regimens not only come with many drawbacks such as increased malignancies, insulin resistance, glucose intolerance and diabetes mellitus [3,4]. Other challenges to HAART efficiency are development of latency and drug resistance as viruses mutate and escape from the drug action [5-8]. Despite isolated stories about cures for HIV infection [9] and a recent modest success in a clinical vaccine trial [10,11], a vaccine that can give total protection and a drug that can give complete cure remain to be designed [12,13].

Immune response to the HIV infection consists of a combination of both humoral and cellular immunity [14,15]. Furthermore, different immune responses can target the same regions of viral peptides. For example, V3-loop peptides of the *Env *gene can be presented by both class I and class II major histocompatibility complex (MHC) molecules and can be recognized by both Cytotoxic T-Lymphocytes (CTLs) and T-Helper cells (Th), as well as by neutralizing antibodies (Ab) (e.g., [16-18]). Likewise, a highly conserved region in the *Gag *gene (287-309 amino acid residues in p24) has been shown to interact with CTL, as well as B and T-Helper cells [19]. This, in turn, implies that escape changes driven by the selection pressure from one type of the host immune response can also lead to escape from a different immune mechanism (e.g., [20]). Recently, epitope vaccines (vaccines that contain synthetic peptides representing epitopes from pathogens) against HIV as well as other viruses such as Influenza have been suggested as a new strategy to avoid the viral escape from the host immune system as well as to counteract development of resistance against drugs [21-24]. While recognition of epitopes by the host immune system and mounting of immune response against pathogen is important in controlling and prevention of infections [25], mutations in the epitope regions can help pathogens to evade recognition by immune receptors and lead to subsequent escape of host immune system [26-28]. Selection by the immune system that promotes amino acid sequence diversification at viral epitopes has been shown to play a significant role in the evolution of different viruses, including HIV-1, SIV, Hepatitis C virus, and the Influenza A virus (e.g., [29-32]).

Based on the type of recognizing receptors, there are three types of epitopes, namely CTL/CD8^+ ^epitopes (CTL), T-Helper/CD4^+ ^epitopes (Th) and neutralizing antibody (Ab) epitopes. Single and multi-epitope vaccines containing CTL, Th and Ab epitopes have been described [33,34]. Inclusion of highly conserved epitopes from different genomic regions in a multi-epitope vaccine has been suggested as a strategy to induce a broader cellular immune response that targets the majority of the virus variants [33,35,36]. However, identification of good vaccine candidates based on the extent of sequence conservation in HIV is a challenging problem, compounded by the fast mutation [37,38] and recombination rates [39-41], overlapping reading frames [42] and overall high degree of sequence divergence among the global HIV-1 population [43].

Recently, we reported a series of highly conserved, co-occurring CTL epitopes from three different genes (*Gag, Pol *and *Nef*) that are frequently found in association with each other and therefore can be considered strong candidates for inclusion in CTL multi-epitope vaccines [44]. However, to further improve the vaccine efficiency, the use of adjuvants capable of inducing a strong cellular response and potentially augmenting these responses should be considered (e.g., [45-48]), including use of multiple types of epitopes [49]. For example, Gram et al. (2009) [49] recently showed that while the use of immune-stimulating adjuvant CAF01 induces strong a CTL response, inclusion of a CD4 T-Helper epitope further improves this CTL response.

Thus, this study was focused on identifying strong associations between different types of epitopes from multiple genes in search of potent multi-epitope vaccine candidates. Our results identified several highly conserved T-Helper epitopes that frequently co-occur with particular highly conserved CTL epitopes and that these epitopes co-occur in the majority of HIV-1 genomes of different subtypes and groups as well as circulating recombinant forms. Here we report 137 unique CTL and T-Helper epitope associations (also referred to as association rules) that involve epitopes from 14 non-overlapping genomic regions from three different genes, namely, *Gag*, *Pol *and *Nef*. Widespread presence of these epitope combinations across highly divergent HIV-1 genomes sampled worldwide, including circulating recombinant forms, coupled with a high degree of evolutionary sequence conservation likely reflective of substantial fitness impacts of escape mutations [50] makes them potent candidates for a multi-epitope vaccine.

## Methods

### HIV-1 genomic sequence data and sequence alignment

HIV-1 sequences in the primary analysis included 90 HIV-1 reference sequences from the 2007 subtype reference set of the HIV Sequence database (Los Alamos National Laboratory (LANL), http://www.hiv.lanl.gov), which included full length genomes containing sequences from all nine protein-coding genes, one sequence per patient (List of sequences, including GenBank accession numbers, is described in the Additional file 1). Amino acid and nucleotide sequence alignments were collected separately for analyses of epitope presence and estimation of nucleotide substitution rates, respectively. These curated alignments were generated using HMMER and verified manually (HIV sequence database by LANL). Further details about sequence alignments and selection of reference sequences are available in the HIV Sequence Database and Leitner et al. (2005) [51], respectively. This reference set was comprised of 47 non-recombinant sequences, including 40 sequences from M group (representing subtypes A1, A2, B, C, D, F1, F2, G, H, J, and K), 7 sequences from N and O groups and 43 recombinant sequences, with approximately 4 representatives for each subtype (Table 1). We used this reference sequence set because it roughly approximates the diversity of each subtype as represented in the database. Inclusion of circulating recombinant forms (CRFs) that are defined as inter-subtype recombinant viruses identified from more than a single patient and spreading epidemically [52,53], allowed us to capture those highly conserved epitopes that are shared with non-recombinant genomes and are also present in the majority of the recombinant reference genomes.

**Table 1:** Overview of HIV-1 sequences used in the analyses.

Type of genome	Group	Subtype	Reference sequences^#^	Non-reference sequences*	**Total (Global HIV-1 population^^^**)
Non - recombinant	M group	A	-	6	6
		A1	4	46	50
		A2	3	-	3
		B	5	158	163
		C	4	350	354
		D	4	32	36
		F1	4	6	10
		F2	4	-	4
		G	4	12	16
		H	3	-	3
		J	3	-	3
		K	2	-	2
		
		M - Total	40	610	650
	
	N group		3	2	5
	O group		4	13	17
	
N & O Total			7	15	22

Non-recombinants - Total			47	625	672

Circulating Recombinant Forms (CRF)			43	263	306

Total			90	888	978

The table shows numbers of HIV-1 sequences of different subtypes among reference sequences and global population used in the analyses.

^# ^Reference sequences used in the primary analyses to identify association rules

* Non-reference sequences were collected from 2008 Web alignment of HIV Sequence database

^^ ^Total number of sequences in the global HIV-1 population used in the analysis

### HIV-1 Epitopes

The sets of CTL, T-Helper and antibody epitopes were collected from the HIV Immunology database (Los Alamos National Laboratory, http://www.hiv.lanl.gov/content/immunology) [54], the most comprehensive curated source of known HIV epitopes [55]. A total of 606 linear epitopes were collected, including 229 CTL epitopes that were described as the "best defined" CTL epitopes and were supported by strong experimental evidence, as defined by Frahm et al., 2007 [56], 296 T-Helper epitopes and 81 antibody epitopes (Table 2, Additional file 2). Because of the challenges in identifying primary sequence elements of structurally conserved discontiguous conformational epitopes (e.g., [57,58]), conformational epitopes were not included in the study. Only the epitopes proven to be immunogenic in human as per the HIV Immunology database were used in this study. The overview of epitope mapping techniques and challenges in epitope identification has been described elsewhere [59,60]. Although CTL and Th epitopes had representation from all nine protein-coding genes, Ab epitopes were absent in the *Vif*, *Vpr*, *Rev *and *Vpu *genes. The majority of the Ab epitopes (75 out of 81) belonged to the *Env *gene, while the *Pol *gene had three and the *Gag*, *Tat *and *Nef *genes had one epitope each [61-65]. It should be noted that because of the high amino acid sequence diversity of the *Env *gene that may differ by as much as 30% between subtypes [43], very few antibody epitopes if at all could be expected to be conserved across a broad range of HIV-1 sequences; thus, in this study we primarily focus on CTL and T-Helper epitopes. Restricting HLA allele(s) for associated epitopes are given in Table 3 as per HIV Immunology database and IEDB http://www.immuneepitope.org/.

**Table 2:** Overview of epitopes used in the analyses.

Gene	Protein	Total no. of epitopes				Highly conserved epitopes*				No of associated epitopes^^^			
		
		CTL^#^	Th	Ab	Total	CTL	Th	Ab	Total	CTL	Th	Ab	Total
*Gag*	p17	18	32	-	50	1	-	-	1	-	-	-	-
	p24	42	88	1	131	8	6	-	14	8	6	-	14
	p2p7p1p6	6	18	-	24	2	-	-	2	2	-	-	2
	
	Total	66	138	1	205	11	6	-	17	10	6	0	16

*Pol*	Gag-Pol	1	-	-	1	-	-	-	-	-	-	-	-
	Protease	8	-	-	8	1	-	-	1	1	-	-	1
	RT	39	20	3	62	12	1	-	13	12	1	-	13
	RT-												
	Integrase	1	1	-	2	1	-	-	1	1	-	-	1
	Integrase	12	11	-	23	5	2	-	7	4	2	-	6
	
	Total	61	32	3	96	19	3		22	18	3	0	21

Vif		9	2	-	11	-	-	-	-	-	-	-	-
*Vpr*		7	6	-	13	-	-	-	-	-	-	-	-
*Tat*		4	6	1	11	-	-	-	-	-	-	-	-
*Rev*		4	5	-	9	-	-	-	-	-	-	-	-
*Vpu*		1	1	-	2	-	-	-	-	-	-	-	-
*Env*		40	82	75	197	-	-	2	2	-	-	1	1
*Nef*		37	24	1	62	2	1	-	3	2	1	-	3

	Total	229	296	81	**606**	32	10	2	**44**	30	10	1	**41**

^# ^CTL epitopes included only the best-defined epitopes as described by Frahm et al. (2007) [56]

* Only those epitopes present in more than 75% of the reference sequences were considered as highly conserved and thus included in the association rule mining. 3 epitopes completely overlapping with other epitopes of same type without amino acid differences were not included.

^^ ^Associated epitopes are epitopes involved in association rules identified with a support value of 0.75 and confidence value of 0.95

**Table 3:** Description of the 44 epitopes used in association rule mining.

Gene	Protein	Non-overlapping genomic regions	Epitope sequence	amino acid coordin ates^@^		Type of epitope	Epitopes involved in 2T-3G^	Non-overlapping genomic regions of 2T-3G epitopes	Number of "unique" association rules each epitope is involved	HLA allele/MAb^$^	Class-I HLA allele supertype association	Alternate HLA allele in case of promiscuous HLA alleles (if known)*	+ if cumulative frequencies of HLA supertype alleles over 10% in the population		
														
				Start	End								European	North American	Sub-Saharan African
Gag	p17	1	WASRELERF^#^	36	44	CTL			0	B*3501, B*5801, B53	B07, B58		+	+	+
	
	p24	2	SPRTLNAWV	16	24	CTL	✓	1	712	B*0702, B42	B07	B42, B39, B81	+	+	+
		3	FSPEVIPMF	32	40	CTL			8	B*57	B58		-	-	-
			EVIPMFSAL	35	43	CTL			1	A*2601, A*6901, B*1501, B*4001	A01, A02, B62, B44		+	+	+
			SEGATPQDL	44	52	CTL			257	A*2601, B*4001	B44, A01	B44	+	+	+
		4	GHQAAMQML	61	69	CTL	✓	2	2752	A*0201, A3, B*1510, B38, B*3901	B27, A03, B07, A02	A03, B38	+	+	+
		5	EPRGSDIAGT	98	107	TH			17	DQ7			+	-	+
		6	IYKRWIILGLNKIVR	129	143	TH			1167				-	-	-
			KRWIILGLNK	131	140	CTL			1541	B*2703, B*2705, B35, DRB1*0101	B27, B07		+	+	+
			KRWIILGLNKIVRMY	131	145	TH			1541	DR1, DRB1*0101, DRB1*0301, DRB1*0405, DRB1*0701, DRB1*0802, DRB1*0901, DRB1*1101, DRB1*1201, DRB1*1302, DRB1*1501, DRB4*0101, DRB5*0101			+	+	+
			WIILGLNKIVRMYSP	133	147	TH	✓	3	1885				-	-	-
			GLNKIVRMY	137	145	CTL	✓		2868	B*1501	B62		-	-	+
			LNKIVRMYSPVSILD	138	152	TH			15				-	-	-
			VRMYSPVSI	142	150	CTL			46	Cw*18			-	-	-
		7	PKEPFRDYV	157	165	TH	✓	4	1866	DQ5			+	-	+

	p2p7p1p6	8	CRAPRKKGC	42	50	CTL			9	B*14	B27		-	+	-
		9	TERQANFL	64	71	CTL			29	B*1801, B*4002, B*4001, B*4402, B*4403	B44		+	+	+

Pol	PR	10	LVGPTPVNI	76	84	CTL			1	A*0201, A*0202, A*0203, A*6802	A02		+	+	+
	
	RT	11	IETVPVKL	5	12	CTL			17	B*4001	B44		+	+	+
		12	GPKVKQWPL	18	26	CTL			6	B*0801, B8	B08		+	-	-
		13	KLVDFRELNK	73	82	CTL	✓	5	1554	A*0301	A03		+	+	+
		14	GIPHPAGLK	93	101	CTL	✓	6	971	A*0301, A11	A03		+	+	+
		15	TVLDVGDAY	107	115	CTL	✓	7	783	A*1101, B*1501, B*3501	B07, A03, B62	B07	+	+	+
		16	NETPGIRYQY	137	146	CTL			30	B*1801, B*4001, B*4002, B*4402, B*4403	B44		+	+	+
			IRYQYNVL	142	149	CTL			31	B*1401	B27		-	+	-
		17	LVGKLNWASQIY	260	271	CTL	✓	8	1117	B*1501	B62		-	-	+
			KLNWASQIY	263	271	CTL	✓		1376	A*3002	A01		-	-	-
		18	WEFVNTPPLVKLWYQ	414	428	TH			65	DRB1*0101, DRB1*0401, DRB1*0405, DRB1*0701, DRB1*0802, DRB1*0901, DRB1*1101, DRB1*1302, DRB1*1501, DRB5*0101			+	+	+
		19	GAETFYVDGA	436	445	CTL			11	A*6802	A03		+	+	+
		20	IVTDSQYAL	495	503	CTL	✓	9	471	Cw*0802			-	-	-
			VTDSQYALGI	496	505	CTL	✓		857	B*1503	B27			+	
	
	RT-Integrase	21	LFLDGIDKA	560	8	CTL	✓	10	557	B*81	B07		+	+	+
	
	Integrase	22	LKTAVQMAVFIHNFK	172	186	TH	✓	11	1172				-	-	-
			KTAVQMAVF	173	181	CTL	✓		1279	B*5701	B07		+	+	+
			KTAVQMAVFIHNFKR	173	187	TH	✓		1041	DRB1*0101, DRB1*0405, DRB1*1101, DRB1*1302			+	+	+
			AVFIHNFKRK	179	188	CTL	✓		631	A*0301, A*1101	A03		+	+	+
			FKRKGGIGGY	185	194	CTL	✓		195	B*1503	B27		-	+	-
		23	VPRRKAKII	260	268	CTL	✓	12	15	B*42	B07		+	+	+
			RKAKIIRDY^#^	263	271	CTL			0	B*1503	B27		-	+	-

Env		24	PIPIHYCAPA^#^	212	221	Ab			0	110.1			-	-	-
		25	IKQI	420	423	Ab			5	E51			-	-	-

Nef		26	VGFPVRPQ	66	73	TH	✓	13	72	DR1, DRw15(2)			-	-	-
			RPQVPLRPM	71	79	CTL			7	B*4201	B07		+	+	+
		27	FLKEKGGL	90	97	CTL	✓	14	258	B*0801	B08	B50	+	-	-

Out of the 44 epitopes included in the association rule mining, 41 were found to be part of association rules. Non-overlapping genomic regions and HLA alleles corresponding to each epitope are also shown.

^# ^Epitopes not involved in any association rule

^@ ^Amino acid coordinates are given with respect to the corresponding gene/protein in the HIV-1 HXB2 reference sequence (GenBank Accession no: K03455)

^ Epitopes involved in association rules with 2 types and 3 genes

^$ ^HLA allele/MAb data given where available (from HIV database & IEDB)

*As per Frahm et al., 2007 [56]

### Inclusion of epitopes in association-rule mining

In order to identify the most broadly represented epitopes, each epitope sequence was aligned with 90 reference sequences and the epitopes present in more than 75% of the reference sequences (i.e., perfect amino acid sequence match in more than 67 sequences) were selected for association rule mining. A total of 47 epitopes, including 33 CTL, 12 T-Helper and 2 antibody epitopes, were present in more than 75% of the reference sequences. Among them one CTL and two Th epitopes were completely overlapping with other epitopes of the same type without amino acid differences and, thus, were excluded from the association rule mining to avoid redundancy (e.g., the CTL epitope from the *Gag *gene VIPMFSAL overlaps with the CTL epitope EVIPMFSAL and is present in exactly the same reference sequences). Epitopes of different types that completely overlap with each other without amino acid differences were also included to take into account multi-functional regions (e.g., the CTL epitope KTAVQMAVF completely overlaps with the Th epitope LKTAVQMAVFIHNFK without amino acid differences). The final set of epitopes consisted of 44 epitopes representing 4 genes, namely, *Gag*, *Pol*, *Env *and *Nef*, and included 32 CTL, 10 Th and 2 Ab epitopes (17 epitopes from *Gag*, 22 from *Pol*, 2 from *Env *and 3 from *Nef*) (Table 2).

### Identification of associated epitopes

To identify frequently co-occurring epitopes of different types, we used association rule mining, a data mining technique that identifies and describes relationships (also referred to as associations or association rules) among items within a data set [66]. Although association rule mining is most often used in marketing analyses, such as "market basket" analysis [67,68], this technique has been successfully applied to several biological problems (e.g., [69-71]), including discovery of highly conserved CTL epitopes [44].

The data on presence and absence of selected 44 epitopes in 90 reference sequences (as described above) was used as the input for the Apriori algorithm [67] implemented in the program WEKA [66,72]. Because of our focus on the highly conserved epitope associations, the minimum support was set at 0.75 to include only association rules present in at least 75% of the reference sequences. The confidence was set very high at 0.95 to generate only very strong associations, i.e., where epitopes co-occur in more than 95% of the sequences, and all generated association rules were exhaustively enumerated and examined. The maximum number of rules identified was set at 100000 to ensure that all association rules above the support and confidence thresholds are captured. Once identified, association rules that involved the same epitopes, but in different order, were "collapsed" into a single "unique" rule (i.e., A occurs with B and B occurs with A are considered the same "unique" rule) [44].

### Epitope-associations in a worldwide set of HIV-1 genomes

To verify whether the association rules identified using a representative reference set reflect associations existing in a worldwide HIV-1 population, we examined a larger set of 978 HIV-1 sequences. This genome set included 888 HIV-1 sequences from the 2008 web alignment of the HIV Sequence database selected to include full-length *Gag*, *Pol *and *Nef *genes for each genome, as well as 90 reference sequences used in the first steps of the analysis. The larger genome set included 650 sequences from the M group, 22 from the N and O groups and 306 recombinant sequences (Table 1, Additional file 3). An epitope-association was considered to be present in a particular genome only if all the epitopes participating in that association rule were present without any amino acid differences.

### Estimation of the nucleotide substitution rates

To assess the extent of sequence divergence of associated epitopes, the number of synonymous nucleotide substitutions per synonymous site (dS) and the number of nonsynonymous nucleotide substitutions per nonsynonymous site (dN) were estimated in 90 HIV-1 reference sequences. Each codon was classified as (i) non-epitope or as epitope region, if the codon was mapped to at least one type of epitope. The epitope regions were further subdivided into (ii) associated epitopes (i.e., epitopes participating in association rules), (iii) non-associated epitopes (i.e., those epitopes that were sufficiently conserved to be included in association rule mining but were not participating in association rules), and (iv) all other, variable, epitopes that were excluded from the association rule mining (i.e., those absent from more than 25% of sequences). Pairwise dN and dS values were estimated using the Nei-Gojobori method with the Jukes-Cantor correction [73]. This simple method was chosen because it is expected to have lower variance than more complicated substitution models [74]. The MEGA4 program [75] was used, and the standard errors were estimated with 500 bootstrap replications.

## Results

### Discovery of epitope associations in 90 HIV-1 reference sequences

**Figure 1:**
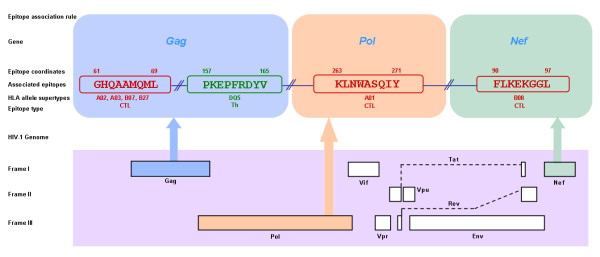
**A "multi-type" association rule involving three CTL and one Th epitope from three different genes, *Gag*, *Pol *and *Nef *in reference to HIV-1 genome**. The corresponding amino acid coordinates (as per HIV-1 HXB2 reference sequence) and HLA allele supertypes recognizing these epitopes are also shown.

Out of 606 epitopes included in the initial analyses, a total of 44 epitope regions, including 32 CTL, 10 Th and 2 Ab epitopes, were present (as a perfect amino acid sequence match) in at least 75% of the 90 HIV-1 reference sequences and thus were included in the association rule mining. Using a high confidence value of 95% allowed us to focus only on the strongest association rules that involve the most frequently co-occurring epitopes. Using this stringent confidence cut-off, a total of 60626 associations involving three types of epitopes belonging to four genes, *Gag*, *Pol*, *Env *and *Nef*, were discovered, of them 6142 association rules were unique combinations of epitopes (Table 4). A total of 41 epitopes that belonged to 27 non-overlapping genomic regions from four genes were found to be involved in these association rules (Table 3). Figure 1 shows an example of an association rule involving four epitopes of two types (CTL and Th) and three genes (*Gag*, *Pol *and *Nef*).

**Table 4:** Distribution of unique association rules according to genes involved in each association rule.

	*Gag *only	*Pol *only	*Nef *only	*Gag-Pol*	*Gag-Env*	*Gag-Nef*	*Pol-Env*	*Pol-Nef*	*Gag-Pol-Nef*	Total*
Association rules with 2 epitopes	46	24	1	55	3	5	1	3	0	138
Association rules with 3 epitopes	104	160	0	768	1	33	0	23	56	1145
Association rules with 4 epitopes	108	135	0	1699	0	29	0	23	104	2098
Association rules with 5 epitopes	73	47	0	1551	0	11	0	4	33	1719
Association rules with 6 epitopes	29	6	0	753	0	2	0	0	3	793
Association rules with 7 epitopes	5	0	0	211	0	0	0	0	0	216
Association rules with 8 epitopes	0	0	0	31	0	0	0	0	0	31
Association rules with 9 epitopes	0	0	0	2	0	0	0	0	0	2

Total	365	372	1	5070	4	80	1	53	196	6142

* There were no epitope associations in the following categories: *Env *only, *Nef-Env, Gag-Pol-Env, Gag-Nef-Env, Pol-Nef-Env, Gag-Pol-Env-Nef*

^$ ^Detailed break-up of number of associations based on epitope type and genes involved is given in additional file 4

**Figure 2:**
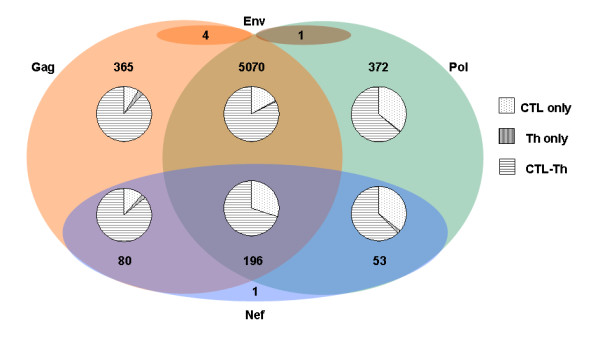
**Relative composition of unique association rules involving multiple genes (*Gag*, *Pol *and *Nef*) and epitope types (Cytotoxic T Lymphocyte (CTL), T-Helper (Th) and antibody (Ab) epitopes)**. The 6142 unique association rules are classified according to the genes that harbor these epitopes. The pie-chart inside each segment represents the division according to the epitope region types involved. The single association rule in *Nef*-only category involved CTL and Th epitopes, while that in *Pol*-*Env *category involved CTL and Ab epitopes. Out of four association rules involving epitopes from *Gag *and *Env*, three belonged to CTL-Ab and one belonged to Th-Ab epitope regions types.

The majority of the unique epitope association rules (cumulatively comprising > 80% of all rules) involved only three to five epitopes, with the largest category comprised of rules with four epitopes (2098 associations), followed by 1719 associations with five and 1145 associations with three epitopes, respectively (Figure 2, Table 4). Notably, a significant number of association rules involved 6 to 8 epitopes (793 associations with six, 216 with seven and 31 with 8 epitopes, respectively). There were only two association rules in which 9 epitopes were involved. More details on number of associations based on epitope type and genes involved are given in Additional file 4. When gene locations were considered, over 82% of the unique epitope associations included epitopes from both the *Gag *and *Pol *genes, followed by 5.9% and 6.1% of associations involving only the *Gag *and only *Pol *genes, respectively. Another 5.4% of unique association rules involved epitopes from the *Nef *gene, of which almost 60% of rules involved three genes, *Gag*, *Pol *and *Nef*, with the remainder distributed mostly between *Gag-Nef *and *Pol-Nef *associations (approximately 24% and 16%, respectively). There were only five association rules that involved epitopes from the *Env *gene. Four of these five were from *Gag-Env *and one from *Pol-Env *associations. Notably, associations with antibody epitopes were limited to these five *Env *association rules, which can partially be attributed to the high degree of sequence divergence among the *Env *sequences that can differ by as much as 30% at the amino acid level [76].

No association rules included all three types of epitopes (CTL, Th and Ab) and four genes (*Gag*, *Pol*, *Env *and *Nef*). However, several "multi-type" association rules comprised of two different epitope types (CTL and Th) and three genes (*Gag*, *Pol *and *Nef*) were discovered (Figure 1, Additional file 5). For example, in the association rule: GHQAAMQML (CTL, *Gag*) - PKEPFRDYV (Th, *Gag*) - KLNWASQIY (CTL, *Pol*) - FLKEKGGL (CTL, *Nef*) (Figure 1), GHQAAMQML, KLNWASQIY and FLKEKGGL are CTL epitopes from the *Gag, Pol *and *Nef *genes, respectively, while PKEPFRDYV is a Th epitope from the *Gag *gene. Overall, there were 137 "multi-type" associations involving epitopes from two types and three genes (2T-3G) among a total of 21 CTL and Th epitopes from the *Gag*, *Pol *and *Nef *genes (Additional file 5). These 21 epitopes can be mapped to 14 different non-overlapping genomic regions (Table 3) and a single association rule is generally spread across 3 to 5 of such regions. Interestingly, even though the association rule with the maximum number of epitopes in a single rule (9 epitopes) involved four non-overlapping genomic regions, it included epitopes from only two genes, *Gag *and *Pol*.

### Epitope-associations in the reference genome are representative of the global HIV-1 population

Presence of association rules discovered in the reference genome set was verified by analyzing a larger worldwide set of 978 HIV-1 genomes (including 888 sequences from the 2008 web alignment and 90 reference sequences from the HIV Sequence database). The *Gag*, *Pol *and *Nef *genes in each sequence were concatenated for the purpose of the analysis, and presence of each association rule (as a complete match of all epitope regions involved) was noted. The results showed that most of the epitope-associations were present in the majority of genomes from the global HIV-1 population. In particular, out of 137 epitope associations involving two different types and three different genes (2T-3G), 134 association rules were present in more than 70% of the HIV-1 genomes (i.e., in > 685 sequences) (Additional file 6). Further, 978 sequences were also analyzed for the presence/absence of 21 individual epitopes participating in the 2T-3G associations. The results revealed that with the exception of a single CTL epitope (VPRRKAKII from the *Pol *gene, present in 65% of the sequences), all other epitopes were present in over 85% of the sequences (Additional file 7).

These results underscore the importance of these 21 highly conserved epitope regions, as reflected by their substantial presence across the global population of HIV-1. Notably, similar pattern of presence with high frequency was observed when the sets of M group sequences (610), as well as sets of recombinant sequences (263), were considered separately. Interestingly, the latter group had these epitopes present in at least 80% of all sequences. On the other hand, only 7 out of the 21 epitopes were present in more than 75% of the sequences when the N and O groups were considered separately, which may reflect both the high degree of sequence divergence between N, O and M groups [43,77], as well as that the majority of epitopes used here were discovered in M group sequences (HIV Molecular Immunology database, http://www.hiv.lanl.gov/content/immunology.

### Associated epitope regions are highly conserved at both amino acid and nucleotide levels

To delineate selective forces affecting the evolution of different genomic regions in HIV-1 genomes, particularly those influencing epitope regions, the number of synonymous substitutions per synonymous site (dS) and the number of nonsynonymous (amino acid altering) substitutions per nonsynonymous site (dN) were estimated in all pairwise sequence comparisons of 90 reference genomes. Each codon was classified into one of four categories, either as (i) non-epitope, or as (ii) associated, (iii) non-associated or (iv) variable epitope regions (see Methods section for details). Overall, in all pairwise sequence comparisons and different categories of epitope regions the number of synonymous substitutions per synonymous site significantly exceeded the number of nonsynonymous substitutions per nonsynonymous site, i.e., dS >> dN (paired t-test, p < 0.001) (Table 5). This indicates that purifying selection plays a significant role in the evolution of HIV including evolution of the epitope regions, which is in agreement with our previous results [44,78,79]. Similar trend of overall dS >> dN (paired t-test, p < 0.001) was also observed when sequences of the N and O groups were considered separately. However, because of the high degree of sequence divergence between the three groups due to their independent origin via separate cross-species transmission events [80-82], we will focus our discussion on the pairwise comparisons of the M group sequences only (including CRFs).

**Table 5:** Nucleotide substitution rates among different epitope and non-epitope regions.

	dN	SE^#^	dS	SE	P-value*
Associated epitopes	0.01062	0.00952	0.20969	0.07091	< 0.001
Non-associated epitopes	0.02387	0.02537	0.24220	0.12666	< 0.001
Not included epitopes	0.10532	0.01277	0.29085	0.04305	< 0.001
Non-epitopes	0.09793	0.01653	0.27329	0.04665	< 0.001

Average pairwise number of nonsynonymous (*d_N_*) and synonymous (*d_S_*) substitutions per nonsynonymous and synonymous site, respectively, estimated at different categories of epitope and non-epitope regions among reference sequences of M group are given.

^# ^Standard errors were estimated with 100 bootstrap replications in MEGA4.

* In pairwise t-tests, the null hypothesis of dS = dN was rejected in all four comparisons.

The average dN and dS values for each category of sites obtained from the pairwise comparisons of the reference sequences from the M group are shown in Table 5. Notably, associated epitopes have significantly smaller dN and dS values than respective dN and dS values at other categories of sites, including non-epitopes (one-way ANOVA and nonparametric Kruskal-Wallis tests, p < 0.001) (see also Additional file 8). While significantly lower dN values at associated epitopes can be attributed to strong purifying selection operating to reduce amino acid diversity at these highly conserved epitope regions, in agreement with our previous results [44,78], the significantly lower dS values indicate that the high degree of sequence conservation exist not only at the amino acid level, but also at the nucleotide level in these associated regions. Notably, when we consider correlations between the levels of synonymous and nonsynonymous sequence divergence from different site categories for the same pair of sequences, relatively strong and statistically significant positive correlations (Pearson correlation coefficient values between 0.67 and 0.77, p < 0.01) exist between dN and dS values for both non-epitope and epitope regions that were not included in the association rule mining, including variable epitopes, but not for associated epitopes. Similar trends are detected using non-parametric correlation (Kendall's tau values between 0.34 and 0.45, p < 0.001). This may be attributed to common factors (such as functional and structural constraints and mutation rate) influencing evolution of these regions, so that the regions with higher dS values are also likely to have higher dN values. On the other hand, the levels of synonymous and nonsynonymous sequence divergence at the associated epitopes have only weak or non-significant correlation both with each other (*r *= -0.14, p < 0.01), as well as with dN and dS values at other regions within the same genomes (see Additional file 9). These results indicate that the lower dS values at the associated epitope regions are not merely the reflection of the overall lower mutation rates, but rather due to specific selective forces preserving nucleotide sequences in these regions, much like purifying selection operating to maintain amino acid sequences at the same epitopes. Although the exact nature of these selection constraints remains to be elucidated, it may be related with the structural constraints at the level of RNA structure, including potential regulatory RNA elements that are yet to be described in the HIV genome [83]. Interestingly, when the number of sites characterized as "structured" and "non-structured" in Watts et al. (2009) [83] study was compared among regions classified as associated epitopes and non-epitopes in this study, the results showed that associated epitope regions tend to harbor a significantly larger proportion of structured than non-structured sites while non-epitopes harbor more non-structured than structured sites (Fisher's exact test, p < 0.05). Because structured regions are expected to be more evolutionary conserved at the nucleotide level to preserve the ability to form secondary or higher-order RNA structures, this is consistent with the overall lower degree of sequence divergence observed among associated epitopes. However, no statistically significant difference was observed when the numbers of structured and unstructured sites were compared between associated epitopes and epitope regions not included in the association rule mining (p > 0.05). This can be attributed to a variety of factors, including that the latter epitope category is a heterogeneous mixture of epitopes that are evolving with different rates under different selection pressures [78,79]. Likewise, as pointed out by Watts et al. (2009) [83], while most structures in their studied HIV-1 model have been well characterized, some structural RNA elements may still require further refinement.

## Discussion

Overall, our results identified a set of strong associations between CTL and T-Helper epitopes that co-occur in the majority of the HIV-1 genomes worldwide and can be considered strong candidates for multi-epitope vaccine and/or treatment targets. There have been several attempts to design multi-epitope vaccines using different strategies for the epitope selection, which is one of the most important steps in a multi-epitope vaccine design. Some studies have suggested computer based epitope prediction methods (e.g., [23,84-86]) for such selection, although accuracy of *in-silico *methods for "prediction of epitopes" is still debated [87]. It has been proposed that a mixture of epitopes representing variable regions or potential escape variants can be used to overcome enormous viral diversity of HIV (e.g., [88,89]). Indeed, some of the hypervariable regions have been shown to be strongly immunogenic eliciting broad cross-subtype-specific responses [90,91]. On the other hand, such highly variable regions may not account for critical functional or structural features of the virus, while epitopes that are highly conserved among different subtypes are likely to be of functional significance and thus less prone to escape mutations [28]. In either case, because of the dynamic nature of intra-patient HIV evolution, the need to achieve a broad immune response can be fulfilled through multi-gene/multi-type approach [1,92], with T-Helper activity playing an important role alongside the CTL response (e.g., [93,94]).

Our results identified several association rules that not only involved two epitope types and three genes, but also were found in the vast majority of HIV-1 genomes analyzed. For instance, the association rule, GHQAAMQML (CTL, *Gag*) - PKEPFRDYV (Th, *Gag*) - KLNWASQIY (CTL, *Pol*) - FLKEKGGL (CTL, *Nef*) (Figure 1) was present in over 83.5% (818 sequences) of the worldwide HIV-1 genomes analyzed. Among these, the epitope GHQAAMQML is restricted by HLA alleles from different supertypes, namely, B07 (B*38), B27 (B*1510, B*3901), A02 (A*0201) and A03 (A*03) while epitopes PKEPFRDYV, KLNWASQIY and FLKEKGGL are recognized by DQ5, A01 (A*3002) and B08 (B*0801) respectively. Notably, many of the associated epitopes harbor other epitopes as sub-sequences that are restricted by yet other set of HLA alleles, thus potentially expanding the breadth of epitope recognition across a broad range of host HLA alleles. For example, in the association rule involving epitopes GLNKIVRMY (CTL, *Gag*) - PKEPFRDYV (Th, *Gag*) - LVGKLNWASQIY (CTL, *Pol*) - FLKEKGGL (CTL, *Nef*), epitope LVGKLNWASQIY includes another epitope, KLNWASQIY, as its sub-sequence. These two epitopes are recognized by alleles from different class I HLA loci, B*1501 (B62) and A*3002 (A01), respectively. This not only increases the potential for recognition population-wide, but also increases the likelihood of this region being recognized within the same individual. Moreover, recent studies have shown promiscuous binding of CTL [95] and Th epitopes [96] in HIV-1, i.e., epitope presentation and T-cell recognition may occur in the context of alternative HLA alleles different from the originally defined HLA alleles. This further enhances potential population coverage for recognition of the associated epitopes. It is worth noting that the involvement of Ab epitopes in association rules described here was quite limited, partly because of the strict presence/absence criteria used in the initial selection of epitopes and association rule mining, as well as the fact that the vast majority of Ab epitopes are located within *Env*, a highly variable genomic region. Only five association rules included a combination of Ab and other epitope types (one Th-Ab, and four CTL-Ab associations). Further, this study did not include conformational epitopes, which form a large number of HIV-1 B cell epitopes. However, inclusion of a suitable Ab epitope should be considered alongside the associated CTL and Th epitopes, although further studies are needed to elucidate mechanisms of epitope association and interaction across different types and to identify the most promising Ab epitope candidates.

Although some individual epitopes have been previously identified as conserved (Additional file 10), lack of uniform criterion for defining conservation and use of different subsets of HIV sequences (and often only few subtypes) in different studies make it difficult to evaluate relative extent of sequence conservation. Thus, our study provides first comprehensive systematic survey of CTL, Th and Ab epitopes that are highly conserved and also co-occur together among all subtypes of HIV-1. There are several advantages of using multiple highly conserved epitopes from different genomic locations, such as those represented by association rules, in HIV vaccine. The highly conserved nature of amino acid sequences of these epitopes, along with the signature of strong purifying selection acting at the nucleotide level of the associated epitopes indicates that these associated regions represent functionally critical genomic regions, thus decreasing the likelihood of successful escape mutations. The reasons behind such conservation remain to be elucidated and may be driven by constraints acting on the viral genome itself or restraints due to virus-host interactions. It is likely that such persistently conserved residues indeed comprise structurally or functionally important elements critical for viral fitness, either due to interactions between the associated regions, or due to their involvement with the "outside" interactors. The latter possibility is indirectly supported by the appearance of compensatory mutations that accompany escape mutations and that may be located elsewhere in the protein sequence (e.g., [97,98]). Further, the structural constraints may also be driven by interactions between regions harboring associated epitopes, direct or indirect. For example, conserved 2T-3G epitopes SPRTLNAWV (CTL) and GHQAAMQML (CTL) from the 5' end of the *Gag *gene are involved in formation of the secondary structure elements, such as helix I and IV, of the p24 capsid protein [99]. Further, of 712 association rules that involve the former epitope, about 41.9% also include the latter epitope (with the remaining rules covering other parts of the HIV-1 genome). Notably, helix I plays an important role in hexamerization of p24 during viral maturation [100] and mutations in that portion of the capsid often give rise to noninfectious viruses [99]. Likewise, the outside positioning of helix IV in the p24 hexameric ring as shown in Figure two of Li et al. (2000) [100] and PDB structure 3GV2 [101] suggests it may participate in protein-protein interactions. It is possible that associated epitopes are involved in RNA-protein interactions as well [102].

An additional advantage of using the associated epitopes is that even if escape mutations are successful at a particular region, the other regions can still be targeted. Moreover, because amino acid changes in these epitope regions are relatively rare, inclusion of these regions in a multi-epitope vaccine can not only provide protection against a broad variety of existing HIV-1 variants including many circulating recombinant forms, but can also offer some protection against the new strains that can arise in the near future. Most importantly, inclusion of epitopes that are immuno-responsive to different arms of the host immune machinery, such as CTL and Th epitope combinations can enable stronger and more efficient immune responses, similar to responses achieved with adjuvant therapies (e.g., [45,48,49,103]).

Thus, our study provides a unique strategy to identify suitable epitope candidates for multi-gene/multi-type vaccines that are both highly conserved across the global HIV-1 population and highly likely to co-occur together in the same viral genome in various HIV-1 subtypes and thus can be simultaneously targeted by multi-epitope vaccines. Some of these conserved epitopes have been included in several recently tested vaccine candidates that showed promising results; however, none have included associated epitopes from all three genes. For example, segments of *Gag, Pol *and *Nef *were included in the recent LIPO-5 lipopeptide vaccine trial that showed T-cell responses in ~50% of vaccines [104], yet it lacked associated epitopes from *Pol *(Additional file 11). Further, because the included epitopes are already derived from the lists of epitopes with experimentally demonstrated immunogenicity in humans, (e.g., the list of "best defined" CTL epitopes by Frahm et al., 2007 [56]), many challenges associated with the accuracy of computational epitope prediction (e.g., [87,105,106]) can be avoided. Moreover, while sequence conservation does not assure that the epitope will be strongly immunogenic (e.g., [107,108]), associated epitopes reported in this study also exhibit a high degree of nucleotide sequence conservation which is not readily identifiable by other tools, such as Epitope Conservancy Analysis Tool [107], making them suitable targets for other types of treatments such as RNA interference [109].

Notably, a high degree of amino acid sequence conservation is not the only factor that influences identification of epitopes as promising candidates. For example, several epitopes included in the association rule mining, namely, PIPIHYCAPA (Ab, *Env*), WASRELERF (CTL, *Gag*) and RKAKIIRDY (CTL, *Pol*), were not involved in any of the 60626 associations that we discovered, showing that high conservation at the amino acid level does not automatically translate into involvement in association rules and that other factors are also at play. While it is likely that associated epitopes are harbored in functionally or structurally important domains and thus experience strong constraints due to protein-protein or RNA-protein interactions [102,110-116], further comprehensive experimental and computational studies are needed to better understand the functional and structural constraints and mechanisms underlying the phenomenon of associated epitopes and evolutionary forces that shape sequence variability of these regions.

## Conclusions

This study provides a comprehensive systematic survey of CTL, Th and Ab epitopes that are both highly conserved and co-occur together among all subtypes of HIV-1, including circulating recombinant forms. Several co-occurring epitope combinations were identified as potent candidates for inclusion in multi-epitope vaccines, including epitopes that are immuno-responsive to different arms of the host immune machinery and can enable stronger and more efficient immune responses, similar to responses achieved with adjuvant therapies. Signature of strong purifying selection acting at the nucleotide level of the associated epitopes indicates that these regions are functionally critical, although the exact reasons behind such sequence conservation remain to be elucidated.

## Abbreviations

Ab: Antibody; AIDS: Acquired Immunodeficiency Syndrome; CRFs: Circulating Recombinant Forms; CTL: Cytotoxic T-Lymphocyte; HAART: Highly Active Anti-Retroviral Therapy; HIV-1: Human Immunodeficiency Virus-1; HLA: Human Leukocyte Antigen; LANL: Los Alamos National Laboratory; MAb: Monoclonal Antibody; RNA: Ribonucleic Acid; Th: T-Helper

## Competing interests

The authors declare that they have no competing interests.

## Authors' contributions

SP did the analyses and wrote the manuscript. HP conceived and coordinated the study and wrote the manuscript. All authors read and approved the final manuscript.

## Supplementary Material

Additional file 1 **90 HIV-1 reference sequences included in the study**. 90 HIV-1 reference sequences (as per 2007 subtype reference set of the HIV Sequence database, Los Alamos National Laboratory) used for the analysis of epitope presence. Click here for file

Additional file 2 **Epitopes included in the study**. 606 epitopes used in the analyses. Only epitopes shown to be immunogenic in human were collected from the HIV Immunology database by Los Alamos National Laboratory. Start and End refer to amino acid coordinates in reference HXB2 genome. Click here for file

Additional file 3 **888 non-reference sequences included in the study**. 888 non-reference sequences that represent global HIV-1 population (90 reference sequences are listed in Additional file 1). Click here for file

Additional file 4 **Number of unique association rules**. Number of unique association rules categorized based on the types of epitopes involved in each association rule. Click here for file

Additional file 5 **137 association rules involving epitopes from two different types and three genes**. 137 association rules involving epitopes from 2 different types (CTL & Th) and three genes (*Gag, Pol *&*Nef*). Each row separated by borders represents a single association rule and each column represents a single non-overlapping genomic region. Red letters denote CTL epitopes, green letters denote Th epitopes. Epitopes on blue background are those from *Gag *gene, while those in tan and green backgrounds are from *Pol *and *Nef *genes, respectively. Click here for file

Additional file 6 **Subtype-wise frequencies of 137 2T-3G association rules**. Subtype-wise frequencies of 137 unique association rules where epitopes from 3 genes and 2 types (2T-3G) are involved. Click here for file

Additional file 7 **Frequencies of 21 epitopes involved in 2T-3G association rules**. Frequencies of 21 epitopes involved in 2T-3G association rules in different groups of HIV-1 sequences used in the analysis Click here for file

Additional file 8 **Box-plot of dN and dS values at different categories of epitopes and non-epitopes**. Box-plot of dN and dS values at different categories of epitopes and non-epitopes. P-values are based on t-tests, comparing respective values among site categories. Click here for file

Additional file 9 **Plots of pairwise dN and dS values between different genomic regions**. Plots of pairwise dN and dS values between (a) Associated epitope regions (b) Variable epitopes that were not included in association rule mining and (c) Non-epitope regions for the M group HIV-1 genome. Noticeably, there were no correlation between dN and dS values from associated epitopes and respective dN and dS values from non-epitope regions or variable epitopes. On the other hand, dN and dS values were correlated between non-epitope regions and variable epitopes. Click here for file

Additional file 10 **List of 41 associated epitopes and references to published papers that reported epitopes as conserved and/or evidence of escape**. List of 41 associated epitopes and respective references that have identified the epitope as conserved and/or provided evidence of escape. It should be noted that the epitope conservation criteria and sets of HIV-1 sequences used to define conserved epitopes varied from study to study. Click here for file

Additional file 11 **List of associated epitopes and whether canonical epitope sequences were included in the recently tested vaccine candidates**. List of associated epitopes and whether or not canonical epitope sequences were included in several recently tested vaccine candidates. Click here for file
