# Supplementary material for: Frequent associations between CTL and T-Helper epitopes in HIV-1 genomes and implications for multi-epitope vaccine designs
Source: BMC Microbiol. 2010 Aug 9;10:212. doi: 10.1186/1471-2180-10-212 (PMC2924856; doi:10.1186/1471-2180-10-212)

Additional file-8: Box-plot of dN and dS values at different categories of epitopes and non-epitopes. P-values are based on t-tests, comparing respective values among site categories.

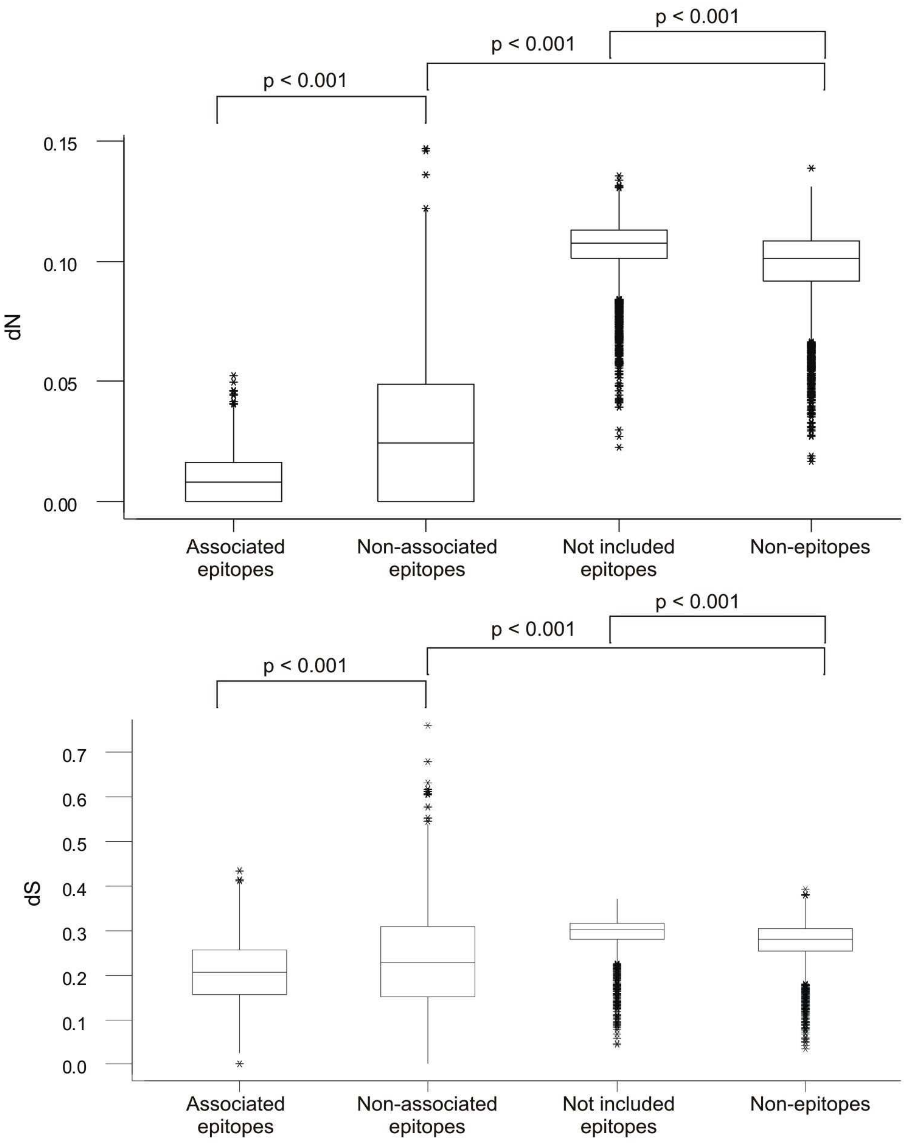

Supplement: Additional file 8 — Box-plot of dN and dS values at different categories of epitopes and non-epitopes. Box-plot of dN and dS values at different categories of epitopes and non-epitopes. P-values are based on t-tests, comparing respective values among site categories. [file 1471-2180-10-212-S8.PDF]
