# Supplementary material for: Frequent associations between CTL and T-Helper epitopes in HIV-1 genomes and implications for multi-epitope vaccine designs
Source: BMC Microbiol. 2010 Aug 9;10:212. doi: 10.1186/1471-2180-10-212 (PMC2924856; doi:10.1186/1471-2180-10-212)
Supplement: Additional file 9 — Plots of pairwise dN and dS values between different genomic regions. Plots of pairwise dN and dS values between (a) Associated epitope regions (b) Variable epitopes that were not included in association rule mining and (c) Non-epitope regions for the M group HIV-1 genome. Noticeably, there were no correlation between dN and dS values from associated epitopes and respective dN and dS values from non-epitope regions or variable epitopes. On the other hand, dN and dS values were correlated between non-epitope regions and variable epitopes. [file 1471-2180-10-212-S9.PDF]

**Additional file-9:** Plots of pairwise dN and dS values between (a) Associated epitope regions (b) Variable epitopes that were not included in association rule mining and (c) Non-epitope regions for the M group HIV-1 genome. Noticeably, there were no correlation between dN and dS values from associated epitopes and respective dN and dS values from non-epitope regions or variable epitopes. On the other hand, dN and dS values were correlated between non-epitope regions and variable epitopes.

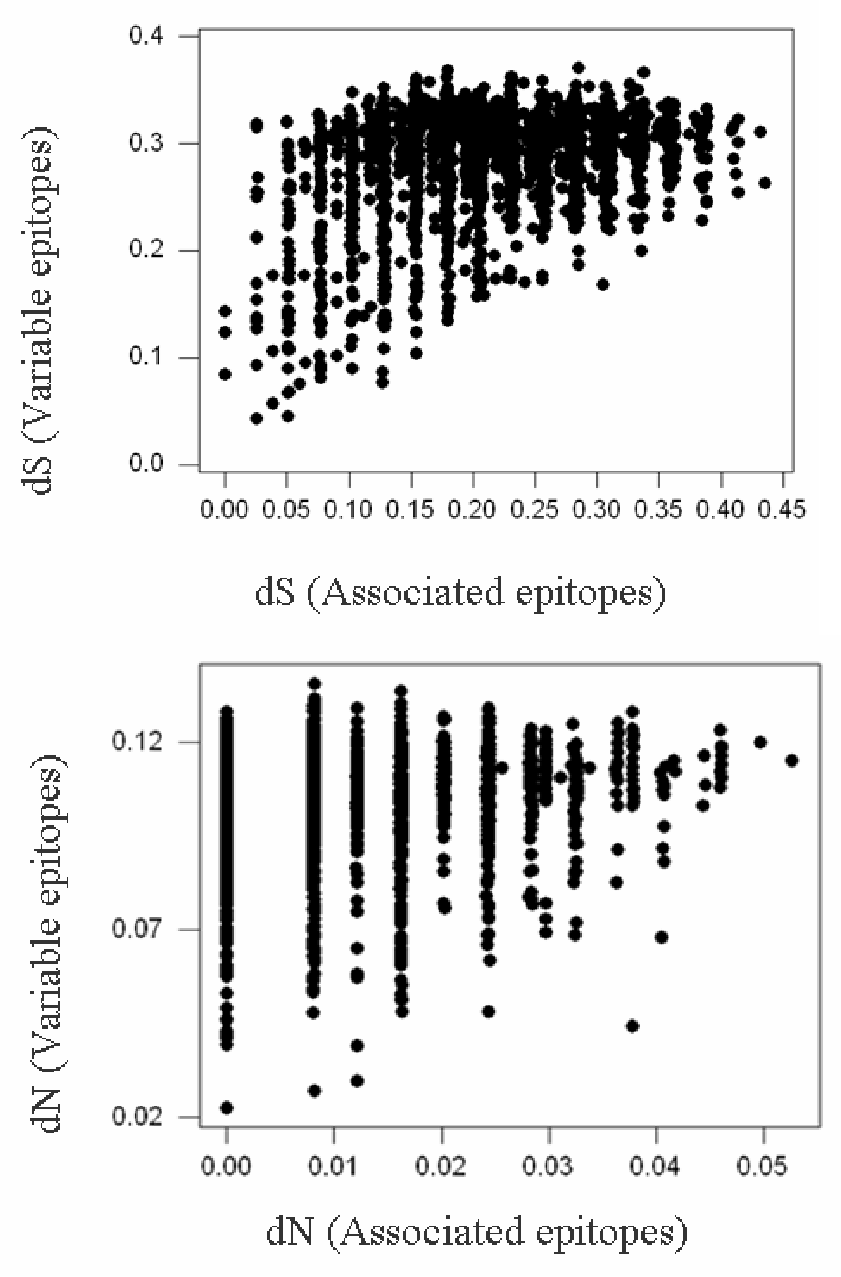

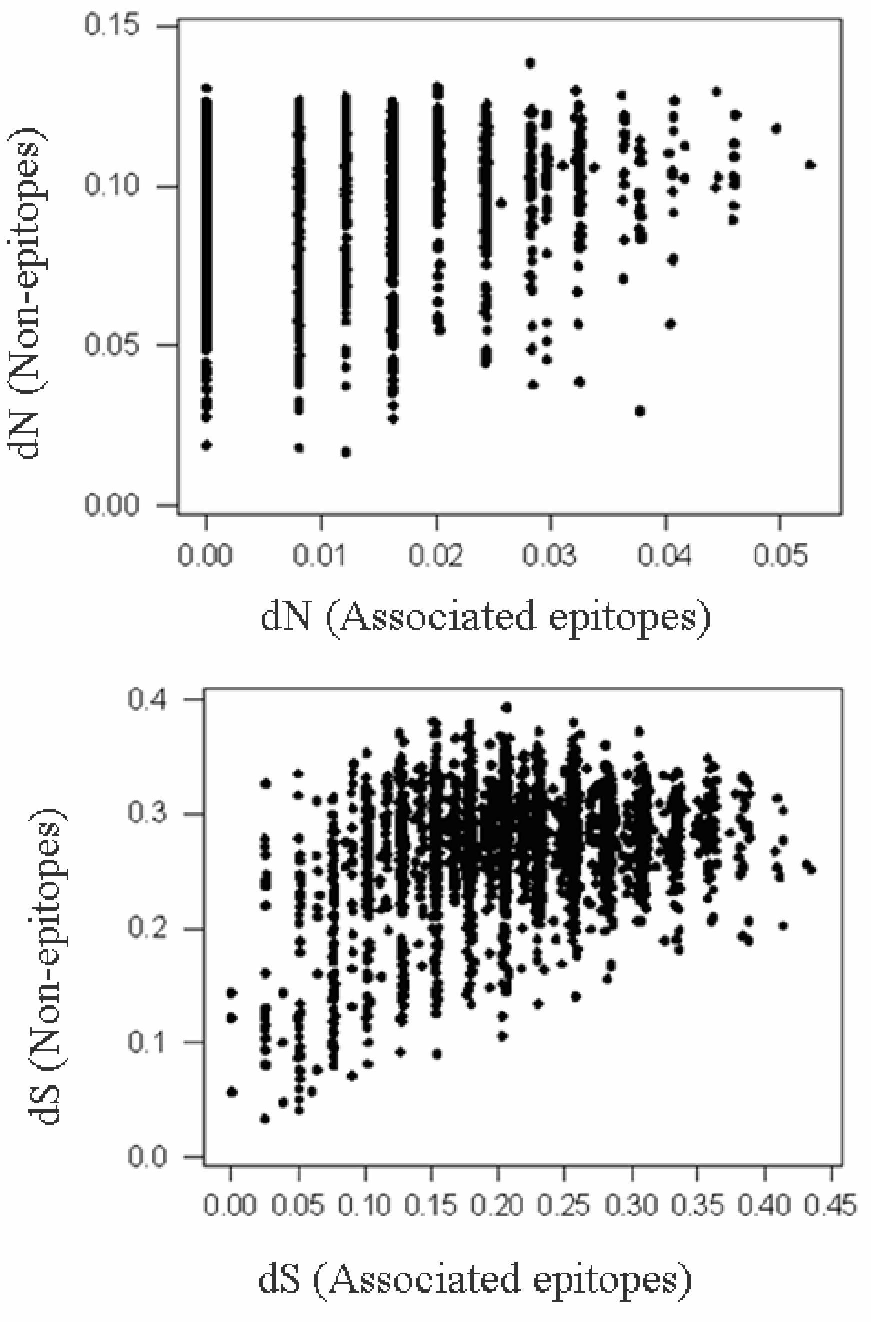
0.15
0.10
0.05
0.00
dN (Non-epitopes)
0.00
0.01
0.02
0.03
0.04
0.05
dN (Associated epitopes)
0.4
0.3
0.2
0.1
0.0
dS (Non-epitopes)
0.00
0.05
0.10
0.15
0.20
0.25
0.30
0.35
0.40
0.45
dS (Associated epitopes)

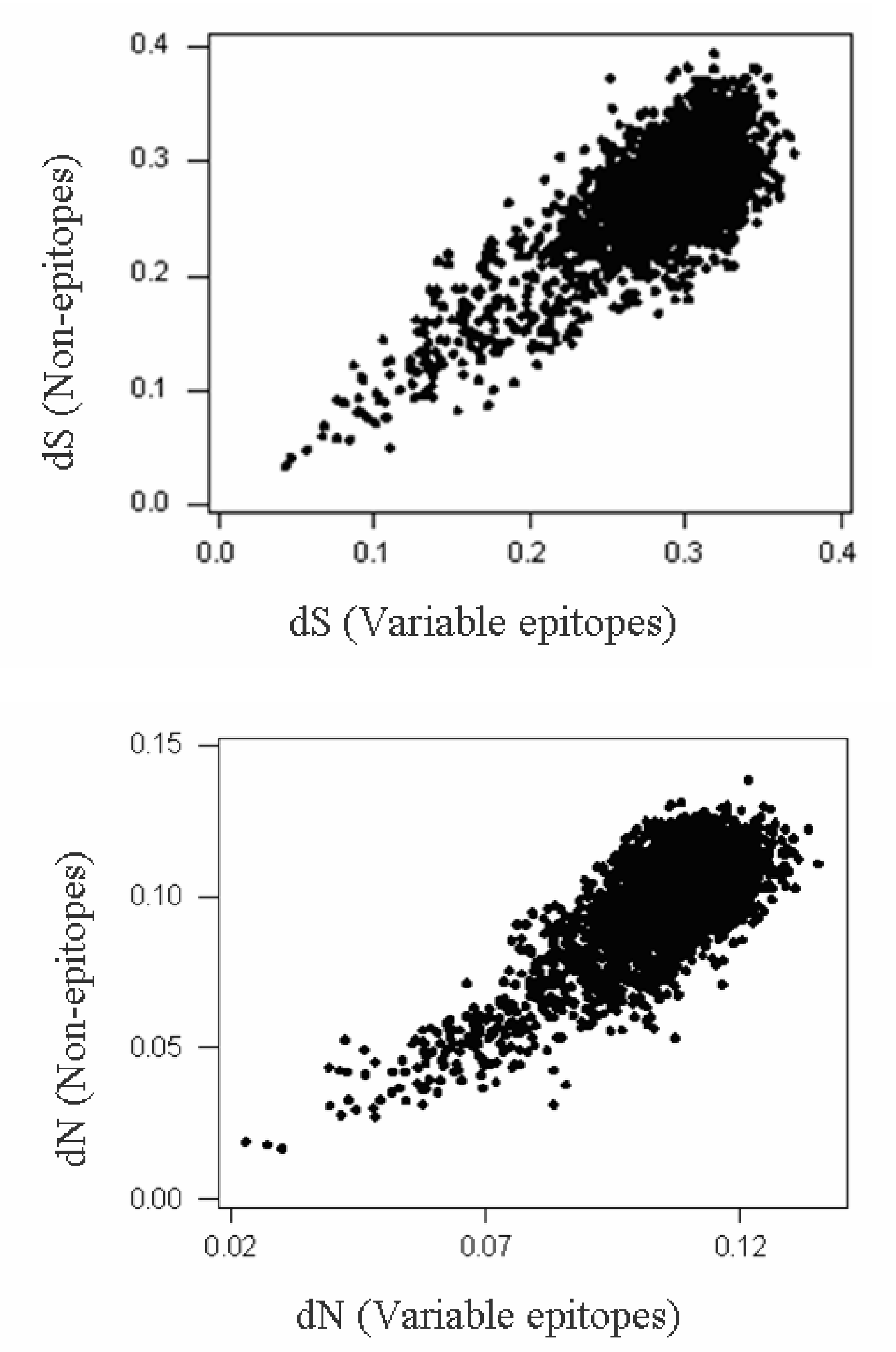

0.4
0.3
0.2
0.1
0.0
dS (Non-epitopes)
0.0
0.1
0.2
0.3
0.4
dS (Variable epitopes)
0.15
0.10
0.05
0.00
dN (Non-epitopes)
0.02
0.07
0.12
dN (Variable epitopes)
